# Physiological mechanism of osmoregulatory adaptation in anguillid eels

**DOI:** 10.1007/s10695-018-0464-6

**Published:** 2018-01-17

**Authors:** Quanquan Cao, Jie Gu, Dan Wang, Fenfei Liang, Hongye Zhang, Xinru Li, Shaowu Yin

**Affiliations:** 1 College of Life Sciences, Key Laboratory of Biodiversity and Biotechnology of Jiangsu Province, Nanjing Normal University, 1 Wenyuan Road, Nanjing, Jiangsu Province 210023 China; 2 Institute of Life Science, Jiangsu University, Zhenjiang, Jiangsu 212000 China

**Keywords:** Anguillid eels, Catadromous migration, Osmoregulation, Ion transport

## Abstract

In recent years, the production of eel larvae has dramatic declines due to reductions in spawning stocks, overfishing, growth habitat destruction and access reductions, and pollution. Therefore, it is particularly important and urgent for artificial production of glass eels. However, the technique of artificial hatching and rearing larvae is still immature, which has long been regarded as an extremely difficult task. One of the huge gaps is artificial condition which is far from the natural condition to develop their capability of osmoregulation. Thus, understanding their osmoregulatory mechanisms will help to improve the breed and adapt to the changes in the environment. In this paper, we give a general review for a study progress of osmoregulatory mechanisms in eels from five aspects including tissues and organs, ion transporters, hormones, proteins, and high throughput sequencing methods.

## Introduction

Dramatic declines in glass eel recruitment of temperate species like the American (*Anguilla rostrata*), European (*Anguilla anguilla*), Japanese (*Anguilla japonica*), and Australian (*Anguilla australis*) eels have raised concerns (Côté et al. 2009; Harrison et al. 2014; Hoyle and Jellyman 2002). The reasons for the declines are unclear but are probably due to reductions in spawning stocks, overfishing, growth habitat and access reductions, pollution, swim bladder, gill parasites and viral infections, global climate change, and the solar cycle (Clevestam et al. 2011; Lin et al. 2010; Parker et al. 2008; Zenimoto et al. 2009). The drastic decline of recruiting wild glass eels has attracted much attention, so it has been increasing an urgent need for technology development in artificial seedlings production (Casselman 2003; Tanaka 2015; Unuma et al. 2012). Since the life history of the eel holds many mysteries, the artificial hatching and rearing of larvae has long been regarded as an extremely difficult task (Okamura et al. 2009; Unuma et al. 2004). The techniques of rearing leptocephalus larvae up to glass eels have been improved significantly in the laboratory, but these are still far from marketization (Celino et al. 2009; Okamura et al. 2014; Tanaka 2003; Tanaka 2014). To understand the artificial hatching and rearing of larvae, it is necessary to study physiological mechanism of osmoregulatory adaptation in anguillid eels.

The anguillid eels, as catadromous species, breed in the sea and migrate to grow near shore or freshwater habitats before returning to spawn in the sea. Therefore, it exhibits a remarkable ability to adapt its physiology as it transfers between seawater (SW, hyperosmotic) and freshwater (FW, hypoosmotic) environments (Maciver et al. 2009). In order to survive, it must completely shift its osmoregulatory system from the one in which excess salt is eliminated and water conserved (in SW), to the one in which the exact opposite is true (in FW). During the migratory process, how the osmotic pressure coordinates with the change of ion concentration to achieve the homeostasis is one of the important tasks in the research of genus *Anguilla* (Aarestrup et al. 2009; Morinière et al. 2002). Corresponding changes in salinity conditions affect the growth and development of eel (Gagnaire et al. 2009; Hu and Duan 2013). Changing ambient conditions will also influence the processing ability of osmotic pressure variation. Thus, it is particularly important to reveal the osmoregulation mechanism of eel and to increase the valuable theoretical basis in the research of genus *Anguilla*.

At present, scholars mainly focused on the osmotic regulation mechanism of anguillid eels and gained some important achievements in different aspects of the research. This article firstly introduces the osmoregulatory organs in the genus *Anguilla*, then presents the details of the osmotic regulation of ion transporters, hormones, proteins, and the transcriptomic and proteomic studies, and finally summarizes and reviews the research progress of osmotic regulation mechanism in recent years.

## Osmoregulatory tissues and organs

Fishes obtain salts from food and ion absorption operating mainly in the gill, kidney, and intestine. (Hiroi et al. 2008; Martinez et al. 2005c; Saglam et al. 2013). These are important osmoregulatory tissues that function to maintain ion and water homeostasis as fish move between environments of wide ranging salinities (Comrie et al. 2001; Kültz 2015; Peh et al. 2009).

In both situations that eels are subjected to a high osmotic stress during migration from FW to SW, one of the major sites of water and ionic exchange between the internal milieu and the external environment are the gills (Lafont et al. 2006). The gill is a major site of passive ion and water movements owing to its large surface area and directly contract with the external environment (Mi et al. 2013; Miyoung 2009). At the same time, the gill is among the most important osmoregulatory organs because of the presence of pavement cells (PVC), mucus cells (MC), and various types of ion-transporting, mitochondrion-rich cells (MRC) (Catches et al. 2006; Girard and Payan 1980; Lai et al. 2015; Mizuhira et al. 1970). MRCs in the gills are the major site of ion absorption and secretion. There is consensus that MRCs maintain transcellular secretion of Cl^−^ and establish the conditions for paracellular electrochemical diffusion of Na^+^, which are important in both FW and SW adaptation (Pelis et al. 2001; Perry 1997; Seo et al. 2013; Utida et al. 1971).

The renal sulfate transport system has dual roles in euryhaline eel, namely, maintenance of sulfate homeostasis and osmoregulation of body fluids (Nakada et al. 2005). The trunk kidney of teleosts is generally composed of numerous nephrons, acting as a functional unit for renal osmoregulation, and the infilling lymphoid tissues (Teranishi et al. 2013). In FW teleosts, the nephron consists of a renal corpuscle and proximal and distal renal tubules, followed by a collecting duct, whereas nephrons in SW teleosts typically lack the distal tubule (Akira et al. 2005; Teranishi 2010). In particular, the kidney plays a major role in euryhaline teleost such as eel as it helps in maintaining body fluid homeostasis during the course of adaptation to different salinities (Li et al. 2015; Martinez et al. 2005a; Teranishi et al. 2013). The kidney is fully by glomerular functions as in any other euryhaline fish (Cliff and Beyenbach 1988; Kenyon et al. 1985; Teranishi 2010). In SW, the euryhaline fish kidney filters plasma at low rates to conserve water, and tubular secretion of electrolytes and fluid contribute significantly to urine formation, serving primarily as the main secretary route for absorbed Mg^2+^, Ca^2+^, and SO_4_^2−^ (Rajagopal and Wallace 2015). While in FW, the kidney filters at high rates and reabsorbs nearly all filtered solutes, thereby producing large volumes of dilute urine. In this way, the osmotic water loads of the FW habitat are excreted (Nishimura et al. 1983; Teranishi et al. 2013).

As for the intestine, it needs to cope with potential dehydration from the hyperosmotic seawater environment (Yuge and Takei 2007). Numerous Na^+^-K^+^-2Cl^−^-cotransporters (NKCCs) are located on the mucosal surface of the intestinal epithelia in SW fish for absorption of these three ions in parallel with water absorption from the intestinal lumen (Schettino and Lionetto 2003; Yuge and Takei 2007). It was well known that water was hardly absorbed in the stomach and intestine, and water absorption predominantly took place in the rectum. For example, the Japanese eel exert hyperosmotic ability as early as during leptocephalus stages, secreting Na^+^ and Cl^−^ through absorbing water from ingested seawater in the rectum (Lee et al. 2012).

Eels sometimes need to adjust saline pressure by nervous system (Hirano et al. 1972; Montero et al. 2010). While neural mechanisms through drinking play an essential role in body fluid balance in marine teleost fish, as the sole means to compensate for osmotic water loss (Hirano et al. 1972; Takei et al. 1979). Drinking behavior is integrated in the brain through several neuropeptides, which may centrally act in a paracrine/autocrine fashion to regulate drinking peripheral hormones through structures devoid of the blood–brain barrier (Takei et al. 1979). In the eel, swallowing is coordinated by the glossopharyngeal and vagus nerves, which originate from the glossopharyngeal-vagal complex (GVC) located in the midbrain–hindbrain area of the brain (Mukuda 2003). The GVC is a motor nucleus controlling swallowing and regulating contraction of the upper esophageal sphincter (UES) muscle via the vagal nerve fibers (Kozaka and Ando 2003; Mukuda and Ando 2003). Therefore, there is a possibility that the relaxin-producing neurons identified in the midbrain and hindbrain send their axons to the GVC and regulate swallowing in eels (Hu et al. 2011). It has been shown that systemic injection of eel atrial natriuretic peptide acts on the area postrema in the eel hindbrain and potently inhibits drinking (Tsukada et al. 2007), and direct injection of adrenomedullins into the cerebral ventricle of eels induced drinking (Ogoshi et al. 2008).

## Ion transporter

The basolateral sodium-potassium ATPase (NKA) concentrates Na^+^ extracellularly, and sodium-potassium-chloride cotransporter (NKCC1) takes up Cl^−^ from the serosal side; NKA concentrates Na^+^ extracellularly, which builds up an electrochemical gradient to excrete Na^+^ via a paracellular pathway while Cl^−^ is excreted via apical CFTR transcellularly due to a lower intracellular voltage (Rajagopal and Wallace 2015). To be specific, NKA maintains the Na^+^ gradient in the cell, which converts the NKCC to cotransport Cl^−^ against its electrochemical gradient. The intracellular Cl^−^ exits the cell via the apical CFTR channel down its electrochemical gradient (Hirose et al. 2003). The movements of essential ion, chloride, and sodium, are effected through a main enzyme, the NKA in these epithelium cells. It also coordinated with other ion channels and transporters, such as the NKCC, the Na^+^/Cl^−^cotransporter (NCC), and the CFTR chloride channel (Lorin-Nebel et al. 2006). Following various mechanisms according to models and salinity, these channels and transporters are involved in the exchanges of both chloride and sodium (NKCC/NCC), or chloride alone (CFTR), either for their uptake (hyper-regulation in fresh water) or excretion (hypo-regulation in seawater) (Hirose et al. 2003; Lorin-Nebel et al. 2006; Marshall 2002; Papadakis et al. 2013; Varsamos et al. 2005). Na^+^ accumulated in the intercellular space exits across the tight junction between neighboring MRCs. In adult eels, the relationship between environmental salinity and branchial NKA has been firmly established at the cellular and molecular level (Wilson et al. 2007). In freshwater teleost fishes, NKA is involved in Na^+^ uptake, although levels of activity tend to be lower than in those found in seawater fishes. Freshwater eels also actively take up Na^+^ and Cl^−^, although at rates significantly lower than other teleosts (Kuroki et al. 2016). In some fishes, *Myoxocephalus octodecemspinosus* and *Anguilla anguilla*, a vacuolar type proton ATPase (V-ATPase) is involved in indirectly driving Na^+^ uptake across the apical membrane via Na^+^ channels by creating a favorable electrochemical gradient (Catches et al. 2006; Harvey 2009; Lorin-Nebel et al. 2013). In *A. anguilla* and *Fundulus heteroclitus*, sodium–proton exchangers (NHE) directly facilitate Na^+^ uptake. The mechanism of Na^+^ uptake in eels has not yet been identified (Cutler and Cramb 2008; Edwards et al. 2005).

The reabsorption of sulfate via the apical Slc13a1 and basolateral Slc26a1 transporters may thus contribute to freshwater osmoregulation in euryhaline eels, via the regulation of circulating sulfate concentration (Nakada et al. 2005). Slc13a1 is an electrogenic Na^+^-dependent sulfate transporter (alternatively called Na^+^-SO_4_^2−^ cotransporter) located in the apical membrane of renal proximal tubule cells and mediates entry of Na^+^-SO_4_^2−^ (Busch et al. 1994). Slc26a1 is sulfate/anion exchanger mediating SO_4_^2−^ efflux across the basolateral membrane in exchange for HCO_3_^−^ (Karniski et al. 1998). Taken together, the eel sulfate transport system, composed of apical Slc13a1 and basolateral Slc26a1, turn out to play dual roles under freshwater conditions: (1) to prevent sulfate loss and maintain sulfate homeostasis and (2) to accumulate and retain relatively high concentrations of sulfate as an osmolyte (Nakada et al. 2005). It explained not only the mechanism of maintaining sulfate homeostasis in freshwater but also why adult eels migrate to freshwater (Chadwick 1989).

## Hormone in osmoregulation

In eels’ reproductive migration, they undergo several physiological and morphological changes, including growth and differentiation of the olfactory system, which is believed to be crucial for navigation during migration and spawning (Westin 1990). In addition, there is an increase in plasma steroid levels, which in teleosts include both estrogens and androgens (Lokman et al. 1998; Sbaihi et al. 2010). The change of the external environment sets off the phenomenon of osmoregulation, which is, in teleosts, under a complex endocrine regulation, involving various hormones such as prolactin, growth hormone, cortisol (Mccormick 2001).

Hormones play an essential role in the regulation of ion and water balance in salinity transfer (Bradshaw and McCormick 2006). In teleosts, one of the major hormones is the corticosteroid that facilitates the processes for the successful acclimation of fish from FW to SW (Aruna et al. 2012b; Balm et al. 1989; Mccormick 2011). Cortisol is the major corticosteroid found in euryhaline teleost fish, with release from inter renal gland being stimulated as fish are transferred from FW into SW (Mccormick 2011; Teles et al. 2013). And it is also often referred to as a SW-adapting hormone, as it is heavily implicated in the ability of fish to maintain water and electrolyte balance when in the SW environment (Aruna et al. 2012a; Kammerer et al. 2010; Tokarz et al. 2013).

The action of this hormone has been reported to include improved water absorptive capacity in both the intestine and the urinary bladder, and increased NKA activity in the gill, which together enable the fish to hyperosmolality in the hypersaline environment (Dantzler 2003). Plasma cortisol levels are known to rise following the transfer of eels from FW to SW, although the hormone’s general role is complex, and its functions include the mediation of the response to several different types of stressors (Fenwick and Forster 1972; Li and Takei 2003). Cortisol infusions in FW eels are able to mimic the effect of SW-acclimation on intestinal aquaporin 1 (AQP1) expression, or in the esophagus of silver eels (Martinez et al. 2005b; Martinez et al. 2005d).

Prolactin (PRL), growth hormone (GH), and somatolactin (SL) are pituitary hormones that control pleiotropic biological functions in teleosts and are originated from a common ancestral molecule (Rand and Swanson 1994 and Celis et al. 2004). Prolactin performs a variety of important functions in vertebrates, is known as the fresh water-adapting hormone in fish, and is also known to be essential for the successful acclimation of fish to FW (Manzon 2002). Elevations in plasma prolactin are positively correlated with salt absorption within the gill and water excretion in the kidney and processes essential for osmoregulation in hypotonic environments (Han et al. 2003; Park et al. 2008; Sudo et al. 2013a). Although this correlation has been known for many years, there is still very little information about the molecular mechanisms responsible for the initiation and maintenance of these physiological processes. Likewise, GH is also known to be a hormone required for SW adaptation, at least during the early stages of acclimation (Sakamoto and McCormick 2006). Although the actions of these hormones are linked to regulation of NKA and a number of other water and solute transporters, the molecular mechanisms responsible for these effects remain unknown. Moreover, GH regulates development and somatic growth and is involved as a hypo-osmoregulatory hormone for seawater adaptation in fish (Sakamoto and McCormick 2006). In contrast, teleost PRL is regarded as a hyper-osmoregulatory hormone for freshwater adaptation (Breves et al. 2011; Eckert et al. 2001; Watanabe et al. 2016). SL is involved in energy mobilization, stress response, calcium metabolism, acidosis, and pigmentation in teleosts, although there is little information of its osmoregulatory functions (Kawauchi and Sower 2006; Sudo et al. 2013b).

In addition, the eel is a euryhaline, migratory species that reverses water and ion regulation when moving between FW and SW environments. One of the most profound changes in regulation is drinking, which increases dramatically when eels are transferred from FW to SW (Hirano 1974). Two major hormones in the eel regulate drinking such as angiotensin II and the natriuretic peptides, and their concentration increased mildly and transiently after SW transfer in blood volume (Okawara et al. 1987; Tsukada and Takei 2006). Relaxins (RLN3) was known as an intrinsic brain peptide that regulates arousal and motivation (Olucha-Bordonau et al. 2003), which also plays an important role in the regulation of drinking in teleost fish (Takei 2008).

## Other proteins in osmoregulation

Water is exchanged through osmosis following ionic gradients, a movement that is facilitated by transmembrane proteins (aquaporins, AQPs) (Giffard-Mena et al. 2007; Lin et al. 2004). It is a major intrinsic protein/aquaporin family of plasma membrane channels, and these aquaglyceroporins are usually capable of transporting small solutes, such as glycerol and urea, as well as water (Gorelick et al. 2006). The effect of AQP3 was widely known on water transport in the kidney of vertebrates in general and of eels in particular, especially in association with migration to the SW environment (Kalujnaia et al. 2007b; Martinez et al. 2005a).

Guanylin, uroguanylin, and lympho-guanylin are members of a family of heat-stable peptides that mediate their actions via the guanylate cyclase receptor isoform termed GC-C (Cutler et al. 2007; Li et al. 2015), which is likely to play a major role in osmoregulation in both freshwater and marine teleosts (Comrie et al. 2001). The major function of guanylin is regulation of water and electrolytes (particularly Cl^−^) transport of the intestine, and uroguanylin also participates in renal handling of water and electrolytes (Yuge et al. 2003). Guanylins are synthesized locally in the intestine and secreted into the lumen to act on the GC-Cs in the apical membrane of eel intestinal cells. Then, intracellular cGMP produces after ligand–receptor interaction activates CFTR and probably induces Cl^−^ and/or HCO_3_^−^ secretion into the lumen as suggested in mammals. The physiological significance of the anion secretion induced by the luminal guanylin/GC-C system on SW adaptation may rival or exceed that of the serosally derived natriuretic peptides in the euryhaline eel (Takei and Yuge 2007).

The members of the cation-chloride-cotransporter (CCC) gene family are most closely associated with the processes of Na and Cl ion absorption in epithelia, such as Na^+^/K^+^/Cl^−^ cotransporter, NKCC2 and the Na^+^/Cl^−^ cotransporter (NCC) (Hoffmann et al. 2007). These two cotransporters are expressed almost exclusively in the thick ascending loop of Henle and the distal convoluted tubule of the kidney respectively and play major roles in NaCl reabsorption during urine formation. The main role of the postulated NKCC2-like Na^+^/K^+^/Cl^−^ cotransporter in eel fish is to absorb ions from imbibed seawater in the intestinal lumen, which result primarily from an osmoregulatory drinking response. The NKCC2-like cotransporter is consequently thought to be present in the apical brush-border membrane of the tall columnar surface epithelial cells of the intestine, where it carries a significant proportion of the sodium, potassium, and chloride ions absorbed from the lumen, into these cells (Ando et al. 2003; Cutler and Cramb 2008). In renal tubules and collecting ducts, Na^+^ and Cl^−^ are reabsorbed from primitive urine through Na^+^/H^+^ exchanger 3 (NHE3) in the proximal tubule, Na^+^-K^+^-2Cl^−^ cotransporter 2 (NKCC2) in the thick ascending limb of Henle’s loop, Na^+^-Cl^−^ cotransporter (NCC) in the distal convoluted tubule, and epithelial Na^+^ channel (ENaC) in the collecting duct (Pochynyuk et al. 2008). Tse et al. demonstrated the effect of osmotic shrinkage that stimulated cell regulatory volume increase (RVI) as well as the expressions of the three important ion transporters: Na^+^/K^+^-ATPase, Na^+^/K^+^/2Cl^−^ cotransporter (NKCC), and Na^+^/H^+^ exchanger-1 (NHE-1) using purified pavement cells (PVCs) (Tse et al. 2006).

## High throughput sequencing method applied in osmoregulation of eel

The application of transcriptomics to the biology of eels can provide a significant increase in basic information making it a powerful tool to enable basic and applied research. High throughput sequencing technologies provide new options to characterize transcriptomes and drive the development of new molecular tools and ultimately leading to a better understanding of the biology of the species (Baillon et al. 2015; Coppe et al. 2010; Gu et al. 2015; Henkel et al. 2012; Minegishi et al. 2012; Natasha et al. 2009; Ohara 2009). With the recent advancements of deep sequencing technology, the generation of transcriptome data is fast and comprehensive, which greatly facilitates the applications of functional genomics (Shokralla et al. 2012; Vera et al. 2008). In 2010, the first transcriptomic study through deep sequencing was performed on gill of *A. anguilla* (Coppe et al. 2010). Utilization of transcriptomic sequencing gave insights into the osmoregulation mechanism, providing transcriptomic view of *A. japonica* catadromous behavior. This annotation provides information of the coding regions of the genes supported by transcriptome. The derived homologous evidences pave the way for phylogenetic analysis of important genetic traits and the improvement of the genome assembly (Liu et al. 2016).

In addition to transcriptome analysis, it is also important to conduct proteomic studies since it is proteins, rather than mRNA, that carry out biological functions (Tomanek 2011). Besides, post-translation modifications such as phosphorylation cannot be determined by mRNA analysis. Recently developed proteomic techniques, such as isobaric tag for relative and absolute quantization (iTRAQ), can provide more reliable quantitative measurements and comparisons among samples than traditional two dimensional electrophoresis (2DE) analysis in Japanese eel (Tse et al. 2015). Moreover, iTRAQ analysis allows identification of more proteins than previous 2-DE proteomics and more reliable quantification of the proteins. Producing a sufficient number of proteins makes it possible to conduct pathway and protein–protein interaction analysis (Wang and You 2012). To be specific, in *Anguilla japonica*, Tse et al. had identified 19 gill proteins that respond to short-term hyperosmotic stress. Together with the protein–protein interaction network analysis, the study revealed the potential importance of the NFKB pathway in osmoregulation (Tse et al. 2013). In *A. marmorata*, Jia et al. detected a large number of differentially expressed proteins by iTRAQ method and a variety of miRNAs with a significant difference between two salinity levels (BW and SW), either in upregulation or downregulation status. These proteins enriched in different GO terms and KEGG pathways suggest the different mechanisms for the acclimation of juvenile eels to brackish water and seawater (Jia et al. 2016; Wang et al. 2015).

By combining the recent technological and methodological advances in transcriptomic and proteomic analysis, this genome wide study aims to identify specific eel gill proteins that play roles in acute FW to SW transfer. Furthermore, the microarray technique for salinity acclimation studies using known genes previously implicated in osmoregulation including prolactin, growth hormone, the Na^+^, K^+^, 2Cl^−^cotransporter, and the Na^+^/K^+^-ATPase; and then also to report some unknown genes, the role of which in osmoregulation remains to be elucidated (Kalujnaia et al. 2007a).

## Conclusions and future perspective

Like zebrafish, the eel belongs to the lower teleostei which are more primitive than pufferfish and medaka, two species of Percomorpha. In this study, we chose eels as a model species because they are euryhaline with excellent osmoregulatory ability, and various experimental techniques had been established in this species for physiological studies (Tsuchida and Takei 1998).

Euryhalinity and environmental stress tolerance are physiological traits that enable eels to complete their life cycle in variable habitats of fluctuating salinity. By contrast, Stenohaline fish, such as common carp, inhabiting osmotically stable environments (the oceans or freshwater lakes and streams) (Kültz 2015). So a wide physiological salinity tolerance range makes eels to have a much stronger trait and complicated function to adapt to high salinity stress. Many ion transporters, hormones, and proteins are involved in salinity stress tolerance, and they compete for the crowded cell interior and for energy resources supporting their synthesis and stabilization.

Some questions are still worth thinking about. Some gene isoforms have tissue-specific or organ-specific expression pattern, which kind of functions they act? There would still more ion channel genes to be cloned in eels compared to mammals. What is more, the application of the knowledge about candidate genes is currently hampered by the lack of understanding of their functions at cell, tissue, and whole-fish levels.

Additionally, no studies examined naturally transfers from a hypersaline condition to either seawater or freshwater. These types of transfers should be explored. Since few studies that examined intraspecific variation in gene expression focused on teleosts, it needs further studies to be expanded in general. Finally, few studies examined gene expression in wild-caught animals, although some were not included because a reasonable approximation of time since transfer could not be estimated. Such a trend calls for better understanding of the biochemical and physiological mechanisms that enable eels to cope with large salinity fluctuations and extreme salinities.
